# Effects of regular breakfast habits on metabolic and cardiovascular diseases: A protocol for systematic review and meta-analysis

**DOI:** 10.1097/MD.0000000000027629

**Published:** 2021-11-05

**Authors:** Zhi-hui Li, Lei Xu, Rao Dai, Li-jie Li, Hao-jie Wang

**Affiliations:** a Department of Orthopedics, Huaihe Hospital of Henan University, Kaifeng,China; b Department of Gastroenterology, Affiliated Hospital of Qinghai University, Xining, China; c Department of Interventional Therapy, Affiliated Hospital of Qinghai University, Xining, China; d Department of Cardiology, Tong Xu County Hospital, Tongxu, China; e Department of Interventional Therapy, Affiliated Hospital of Qinghai University, Xining, China.

**Keywords:** cardiovascular diseases, meta-analysis, metabolic diseases, mortality

## Abstract

**Background:**

Breakfast, which is considered as an important meal of the day, is being ignored by an increasing number of people as the pace of modern life accelerates. Although a large number of previous studies have reported the relationship between skipping breakfast and type 2 diabetes mellitus, most of them were cross-sectional studies. It remains unclear how skipping breakfast affects such specific cardio-metabolic diseases as hypertension, strokes and hypercholesterolemia.

**Methods:**

The protocols and reports of this meta-analysis are based on a meta-analysis of observational studies in epidemiological guidelines (MOOSE). Relevant studies were systematically retrieved from PubMed, Embase, Web of Science and the Cochrane Library, and were restricted to English from the inception to May 10, 2019. All the results were obtained by RRs, and outcomes of interests should include the occurrence of cardiovascular and metabolic diseases.

**Results:**

Fourteen cohort studies in total were eventually included. Compared with people having breakfast frequency_***≦3times/week***_, those with a frequency_***>3 times/week***_ have reduced the risk of type 2 diabetes mellitus, obesity, Metabolic Syndrome, Low high-density lipoprotein cholesterolemia, Cardiovascular Diseases, cardiovascular Mortality, hypertension and strokes, with (RR = 0.8 [95% CI: 0.7–0.91], *P* = .142, *I*^2^ = 37.6%), (RR = 0.74 [95% CI: 0.59–0.94], *P* < .001, *I*^2^ = 89%), (RR = 0.86 [95% CI:0.75–0.99], *P* = .512, *I*^2^ = 0%), (RR = 0.75 [95% CI:0.61–0.93], *P* = .643, *I*^2^ = 0%), (RR = 0.87 [95% CI:0.81–0.93], *P* = .479, *I*^2^ = 0%), (RR = 0.63 [95% CI:0.51–0.78], *P* = .396, *I*^2^ = 0%), (RR = 0.92 [95% CI:0.86–0.98], *P* = .419, *I*^2^ = 0.7%), and (RR = 0.89 [95% CI:0.79–0.99], *P* = .238, *I*^2^ = 29%), respectively.

**Conclusions:**

A regular daily breakfast habit benefits the cardio-metabolism to a great extent, reducing the risk of Cardiovascular Diseases, type 2 diabetes mellitus, obesity, hypertension, strokes, Metabolic Syndrome, cardiovascular Mortality, Low high-density lipoprotein cholesterolemia, and Abdominal obesity, while it is not significantly related to hypercholesterolemia and coronary heart disease regardless of gender. Nevertheless, skipping breakfast once a week may greatly reduce the benefits of cardio-metabolism. Therefore, public institutions should promote and encourage citizens to cultivate regular daily breakfast habits.

## 1 Introduction

With the acceleration of the pace of modern life, breakfast, which is regarded as an important meal of the day, is being ignored by more and more people, which seemingly as a universal behavior, may have negative effects on your health.^[1,2]^ There are various reasons for skipping breakfast. For office workers, they may not have enough time to eat breakfast. For students, they may lack a good breakfast habit. As for obese patients, they may have a poor appetite or must limit their energy intake. As a matter of fact, regular breakfast habits can keep us energetic in work and study. In addition, eating breakfast can increase satiety, thereby reducing overeating later in the day to restrict weight gain.^[3]^ Recently, several studies have shown that skipping breakfast increases the risk of obesity,^[4]^ hypertension,^[5]^ hypercholesterolemia (HC),^[6]^ type 2 diabetes mellitus (T2DM),^[7]^ metabolic syndrome (MetS),^[8]^ coronary heart disease (CHD),^[9]^ and cardiovascular mortality (CVM).^[10]^ Conversely, can regular breakfast habits reduce cardiovascular and metabolic diseases?

Most of the previous dietary studies have focused on dietary components and combinations,^[11–14]^ such as dietary fat, cereals, and the Mediterranean diet patterns, while few pay attention to the effects of daily eating behavior on cardiovascular diseases (CVD) and metabolic diseases (MetD). Besides, although a large number of previous studies have revealed the relationship between skipping breakfast and diabetes, most of them were cross-sectional ones.^[14–17]^ In this case, it is still unclear how skipping breakfast affects some other specific cardio-metabolic diseases. Therefore, the purpose of this meta-analysis is to systematically investigate the association between regular breakfast habits and cardio-metabolic diseases, and to update the epidemiological evidence so as to better serve public health and health promotion activities.

## 2 Methods

### 2.1 Literature search and study selection

The protocols and reports of this meta-analysis rely on a meta-analysis of observational studies in epidemiological guidelines (MOOSE).^[18]^ Relevant studies were systematically retrieved (Zhi-hui and Xu) from PubMed, the Embase database and the Cochrane Library, and were restricted to English from the inception to May 10, 2019. Furthermore, the manual retrieval of the library was carried out. To ensure a comprehensive search, three sets of medical subject headings (MeSH) including “breakfast,” “cardiovascular diseases,” and “metabolic disease” were used. Generally, the Boolean operator “And” is employed between the two sets of keywords, and the “Or” is adopted within each group. Specifically, the first step was to use Boolean operator “Or” to combine the two sets of Mesh (cardiovascular diseases and metabolic diseases) and their corresponding synonyms, and then the Boolean operator “And” was applied to combine the Mesh of breakfast and its related synonyms. Besides, previous meta-analyses and systematic reviews were reviewed for comprehensive inclusion in the study. See Appendix 1 (Supplemental Digital Content***)*** for a detailed search strategy.

According to PICOS criteria, the inclusion criteria for the study were as follows:

1. The study population was the general population, aged > 18 years, with no previous history of metabolic or cardiovascular disease.
2. Regular breakfast eaters as the intervention group.
3. Irregular or non-breakfast eaters as the control or reference group.
4. Outcomes of interests should contain the occurrence of cardiovascular and metabolic diseases.
5. The study type was limited to cohort studies or randomized-controlled trials.
6. Studies were provided with available maximum adjustment odds ratios (ORs), risk ratios (RRs), hazard ratios (HRs), and the corresponding 95% confidence intervals (CIs).
7. Language was limited to English.

Meanwhile, the exclusion criteria were shown below:

1. The study subjects were people who previously had CVD or MetD.
2. The exposure of the studies was non-breakfast frequency, such as specific-ingredients in the food spectrum.
3. Cross-sectional studies, reviews, case reports, conference abstracts, and letters were excluded.
4. The outcomes of studies were non-cardiovascular and non-metabolic diseases.
5. The related ORs, RRs or HRs and the corresponding 95% CIs of studies could not be obtained.
6. The language of studies was non-English.
7. For duplicated publications, the longest follow-up data or the largest number of population would be included for analysis.

### 2.2 Data extraction and quality assessment

The following data, like the first author, the year of publication, the country, the duration of follow-up, the mean age, females, the sample size, exposure assessment, CVD/MetD assessment, intervention (breakfast frequency), control (breakfast frequency), outcomes, and main findings, were extracted with a unified data list made by two independent reviewers (Li and Liu). Any disagreements and disputes in the process of data extraction shall be resolved through negotiation. Besides, the Newcastle-Ottawa scale (NOS)^[19]^ was adopted to assess the quality of the study, with a total score of 9. To be specific, it is believed that studies with a NOS score over 6 stars are of high quality, while those with a NOS score below 6 stars are considered as low-quality ones.

### 2.3 Statistical analysis

Our primary outcomes would focus on the risk of cardiovascular and metabolic diseases, as well as specific diseases such as hypertension, type 2 diabetes mellitus, obesity, and strokes. Broadly speaking, the HR was equivalent to the RR, and was thereby directly considered to be the RR.^[20]^ If necessary, use the following formula to convert ORs to RRs and calculate the corresponding 95% CIs. Relative risk=odds ratio/[(1 − P0) + (P0 × odds ratio)], where P0 indicated the incidence of outcomes in the unexposed group.^[21]^ Then, convert the standard error (SE) of the RR with the following formula: SElog(relative risk) = SElog(odds ratio) × log(relative risk)/log(odds ratio).^[22]^ In addition to that, if P0 was rare (*P* < .05), ignore the differences among various measures of relative risk (e.g., OR, RR, and HR).^[23]^ All the results were shown by RRs. As the reference groups of each study were not identical, the frequency of breakfast was divided into the following five groups: ≤3 times/week, >3 times/week, 4 to 6 times/week, 0 to 6 times/week, and 7 times/week, to ensure homogeneity and effective consolidation of data. This group of one cohort 25 study was 0 to 4, and it was roughly classified as 0 to 3 for the effective combination of data. Besides that, the group with the lowest breakfast frequency was unified into the reference group through the Excel macro file made by Hamling et al^[25]^ based on Greenland and Longnecker's theory.^[26]^ In general, most studies reported specific breakfast frequency, but a few articles did not mention breakfast frequency. We contacted the original authors, and if relevant information was still not available, we would classify “Eats breakfast (yes) or eating breakfast” as 7 times per week and “Eats breakfast (No) or skipping breakfast” as 0 time per week. Besides, if the subject was specifically classified according to gender and age, we would tend to consider it as two studies. Statistical heterogeneity was assessed by using *I*^2^ statistics, where 25%, 50%, and 75% of *I*^2^ values represented low, medium and high heterogeneity, accordingly.^[27]^ If *I*^2^ was ≤50%, the fixed-effect model would be adopted. Otherwise, the random effect-model would be adopted. If the confidence intervals of 95 are on one side of 1, we think there is a significant difference. Meanwhile, Begg's test was performed to evaluate the potential publication bias,^[28]^ and subgroup and sensitivity analysis were used to explore sources of potential heterogeneity. All data analyses were conducted by Stata SE12.0 software.

### 2.4 Ethical statement

The data analyzed in this study were extracted from previously published studies, and therefore ethical approval was not necessary.

## 3 Results

**Figure 1:**
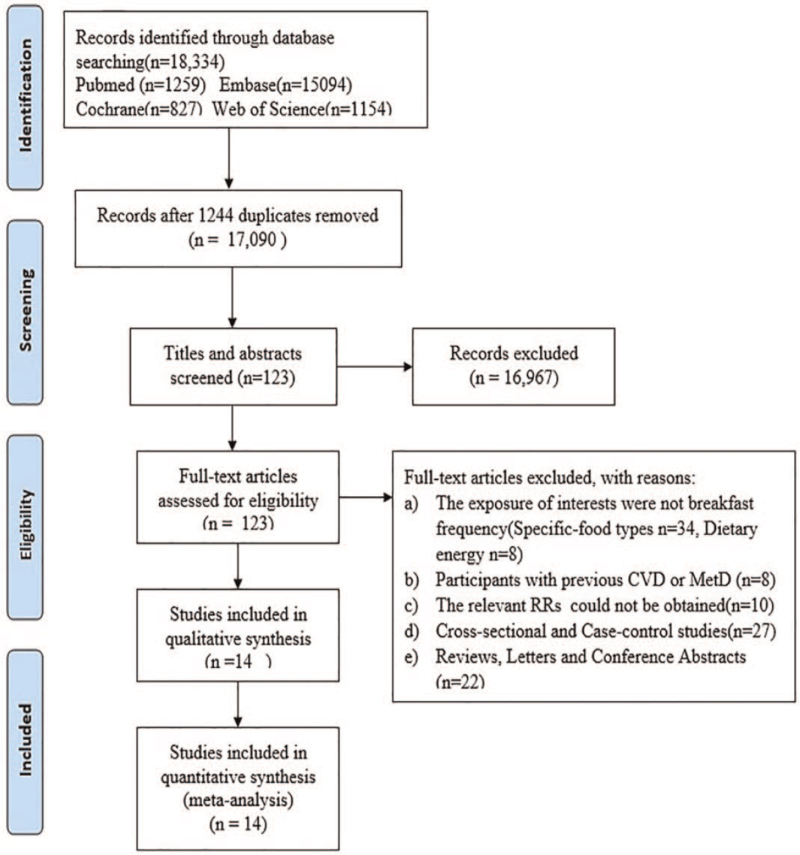
The retrieval flow chart.

A total of 18,334 studies were identified from the four electronic databases, namely PubMed, Embase, the Cochrane Library, and Web of Science, as shown in Figure 1. No additional studies were added by manual search. Of the 18,334 studies, 17,090 were retained after 1,244 duplicated studies were excluded, and 16,976 unrelated studies were abandoned by screening titles or abstracts. After a detailed review on the full text of 123 studies, 108 studies were eliminated for the following specific reasons:

1. The exposure of interest was not breakfast frequency (specific-food types n = 34, dietary energy n = 8).
2. Participants had a history of CVD or MetD (n = 8).
3. The relevant RRs could not be obtained (n = 10).
4. Cross-sectional and Case–control studies were excluded (n = 27).
5. Reviews, Letters, and Conference Abstracts were excluded (n = 22).

Finally, 14 cohort studies^[7,9,24,29–39]^ were involved, and among them, 6 studies showed the relationship between breakfast frequency and T2DM; 5 studies revealed the risk of obesity; 4 studies reported the risk of hypertension; 3 studies indicated the risk of MetS and abdominal obesity, while 2 pointed out the risk of CVD, strokes, hypercholesterolemia, CVM, Low levels of high-density lipoprotein cholesterol (LHDL-c) and CHD. Table 1 displayed the detailed characteristics of the study. The quality assessment of the included studies was shown in Table 2. All the studies scored over 6 stars were featured with high quality.

**Table 1:** Detailed characteristics of the 14 cohort studies included in this meta-analysis.

Author, year	Country	Follow-up, year	Study design	Sample size, female (%)	Age, year	Exposure assessment	CVD/MetD assessment	Intervention (breakfast frequency)	Control (breakfast frequency)	Outcome	Main findings
Jaaskelainen et al^[24]^ 2012	Finland	1986–2002	Cohort study	6247, 51%	16	Q	International Diabetes Federation paediatric definition	7	0∼4	Obesity	Among 16-year-olds, the five-meal-a-day pattern was robustly associated with reduced risks of overweight /obesity in both genders and abdominal obesity in boys.
										Hypertension	
										HC	
										A-obesity	
Sugimori et al^[29]^ 1998	Japan	1976–1991	Cohort study	2573, 28%	46.6	Q	FBS≥ 110 mg/dL or DT	1∼7	0	T2DM	For females, breakfast skipping is positively associated with incidence of T2DM.
Uemura et al^[30]^ 2014	Japan	2002–2011	Cohort study	4631, 22.3%	47.6	Q	FBG≥126 mg/dL, medical record	1∼2	0	T2DM	Breakfast skipping is positively associated with incidence of T2DM.
								3∼5			
								6			
								7			
Byrne et al^[31]^ 2016	USA	2003–2012	Cohort study	10,248, 68.1%	41.2	Q	Concise Health Risk Assessment	2∼3	0–1	T2DM,	Top priorities for workplace health promotion should include low-fat diet, aerobic exercise, nonsmoking, and adequate sleep.
								4∼6		CVD	
								7		Obesity	
										Stroke	
										Hypertension	
										HC	
Odegaard et al^[7]^ 2013	USA	1992–2011	Cohort study	3598, 55.7%	32.0	Q	BMI ≥ 30 kg/m^2^	4∼6,	0–3	T2DM	Daily breakfast intake is strongly associated with reduced risk of a spectrum of metabolic conditions.
							SBP ≥ 140 mm Hg	7		Obesity	
							DBP ≥ 90 mm Hg			Hypertension	
							NCEP-ATP III			MetS	
							FBG ≥ 6.99 mmol/L			A-obesity	
							2 h PG ≥ 11.1 mmol/L				
Cahill et al^[32]^ 2013	USA	1992–2008	Cohort study	51,529, 0	58.6	Q	Medical records or autopsy reports	7	0	CHD	Eating breakfast was associated with significantly lower CHD risk in this cohort of male health professionals.
Mekary et al^[33]^ 2013	USA	2002–2008	Cohort study	121,700, 100%	67.2	Q	American Diabetes Association Criteria	7	0–6	T2DM	Irregular breakfast consumption was associated with a higher T2D risk in women
Rong et al^[9]^ 2019	China	1988–2011	Cohort study	6550, 52%	53.2	Household Interview	ICD-9	1∼3	0	CVM	Skipping breakfast was associated with a significantly increased risk of mortality from CVD.
							ICD-10	4∼6			
								7			
Wennberg et al^[34]^ 2014	Sweden	1981–2008	Cohort study	889, 52.2%	43	Q	International Diabetes Federation	7	0	MetS	Poor breakfast habits in adolescence predicted the metabolic syndrome in adulthood.
										Hypertension	
										LHDL-c	
										A-obesity	
Yokoyama et al^[35]^ 2016	Japan	1988–2009	Cohort study	83,410, 59%	40–79	Q	ICD-10	7	0	CVM	Our findings showed that skipping breakfast is associated with increasing risk of CVM.
							ICD-9				
Kubota et al^[36]^ 2016	Japan	1995–2010	Cohort study	82,772, 53.2%	56.5	Q	The criteria of the National Survey of Stroke	7	0	CVD	The frequency of breakfast intake was inversely associated with the risk of stroke
										Stroke	
										CHD	
Mekary et al^[37]^ 2012	USA	1992–2008	Cohort study	29,206, 0%	58.1	Q	American Diabetes Association Criteria	7	0	T2DM	breakfast consumption was inversely associated with T2D risk in men
Wijtzes et al^[38]^ 2016	The Netherlands	2y	Cohort study	5913, 50.3%	6	Q	International Obesity Task Force	7	0–6	obesity	Breakfast skipping at age 4 years is associated with a higher percent fat mass at age 6 years
Kim et al^[39]^ 2015	Korea	2001–2006	Cohort study	1228, 100%	46.9	Household Interview	NCEP-ATP III	7	0	MetS	Implications include the need for stronger emphasis on weight control before midlife and experiencing menopause

A-obesity = Abdominal-obesity, CVD = Cardiovascular Diseases, CVM = cardiovascular Mortality, DBP = diastolic blood pressure, DT = diabetic therapy, FBS = fasting blood sugar, HC = Hypercholesterolemia, ICD = International Statistical Classification of Diseases, LHDL-c = Low HDL cholesterolemia, MetD = Metabolic Diseases, MetS = Metabolic Syndrome, NCEP-ATP III = National Cholesterol Education Program Adult Treatment Panel III criteria, PG = postchallenge glucose, Q = Questionnaire, SBP = systolic blood pressure.

**Table 2:** Quality assessment of the 14 included studies.

	Selection						Outcome			
Study (author, year)	Exposed cohort	Nonexposed cohort	Ascertainment of exposure	Outcome of interest	Comparability		Assessment of outcome	Length of followup	Adequacy of follow-up	Total
Jaaskelainen et al (2012)	^∗^	^∗^	^∗^	^∗^	^∗^	^∗^	^∗^	^∗^		8
Sugimori et al (1998)	^∗^		^∗^	^∗^	^∗^		^∗^	^∗^	^∗^	7
Uemura et al (2014)	^∗^	^∗^	^∗^	^∗^	^∗^		^∗^	^∗^	^∗^	8
Byrne et al (2016)	^∗^	^∗^	^∗^	^∗^	^∗^		^∗^	^∗^	^∗^	8
Odegaard et al (2013)	^∗^		^∗^	^∗^	^∗^		^∗^	^∗^	^∗^	7
Cahill et al (2013)	^∗^	^∗^	^∗^	^∗^	^∗^		^∗^	^∗^	^∗^	8
Mekary et al (2013)	^∗^	^∗^	^∗^	^∗^	^∗^	^∗^	^∗^	^∗^		8
Rong et al (2019)	^∗^	^∗^	^∗^	^∗^	^∗^	^∗^	^∗^	^∗^		8
Wennberg et al (2014)	^∗^	^∗^	^∗^	^∗^	^∗^		^∗^	^∗^		7
Yokoyama et al (2016)	^∗^	^∗^	^∗^	^∗^	^∗^	^∗^	^∗^	^∗^		8
Kubota et al (2016)	^∗^	^∗^	^∗^	^∗^	^∗^		^∗^	^∗^	^∗^	8
Mekary et al (2012)	^∗^	^∗^	^∗^	^∗^	^∗^	^∗^	^∗^		^∗^	8
Wijtzes et al (2016)	^∗^	^∗^	^∗^	^∗^	^∗^	^∗^	^∗^	^∗^		8
Kim et al (2015)	^∗^	^∗^	^∗^	^∗^	^∗^	^∗^	^∗^	^∗^	^∗^	8

∗ 1 point. Total, total score.

### 3.1 Meta-analysis

#### 3.1.1 T2DM

**Figure 2:**
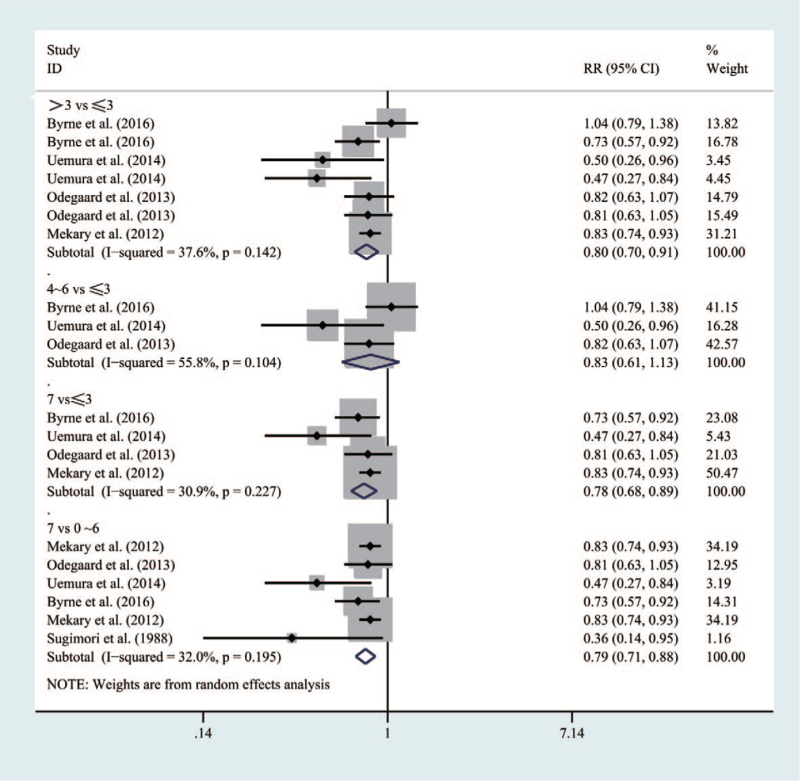
Forest map of the relationship between breakfast frequency and the risk ofT2DM.

Figure 2 shows that six studies involved 171,956 participants in the current meta-analysis. Compared with people who had a breakfast frequency_***≤3times/week***_, those with the frequency_***>3 times/week***_ and frequency_***7times/week***_ had a lower risk of T2DM (RR = 0.8 [95% CI: 0.7–0.91], *P* = .142, *I*^2^ = 37.6%) and (RR = 0.78 [95% CI: 0.68–0.89], *P* = .227, *I*^2^ = 30.9%), respectively. Besides, compared with people who had a breakfast frequency_***0∼6times/week***,_ those with a frequency_***7times/week***_ would significantly decrease the risk of inducing T2DM (RR = 0.79 [95% CI: 0.71–0.88], *P* = .195, *I*^2^ = 32%). However, risk for T2DM of those with a breakfast frequency4 to 6times/week would not be lower than that of those with a frequency ≤ 3 times/week (RR = 0.83 [95% CI: 0.61–1.13], *P* = .104, *I*^2^ = 55.8%).

**Figure 3:**
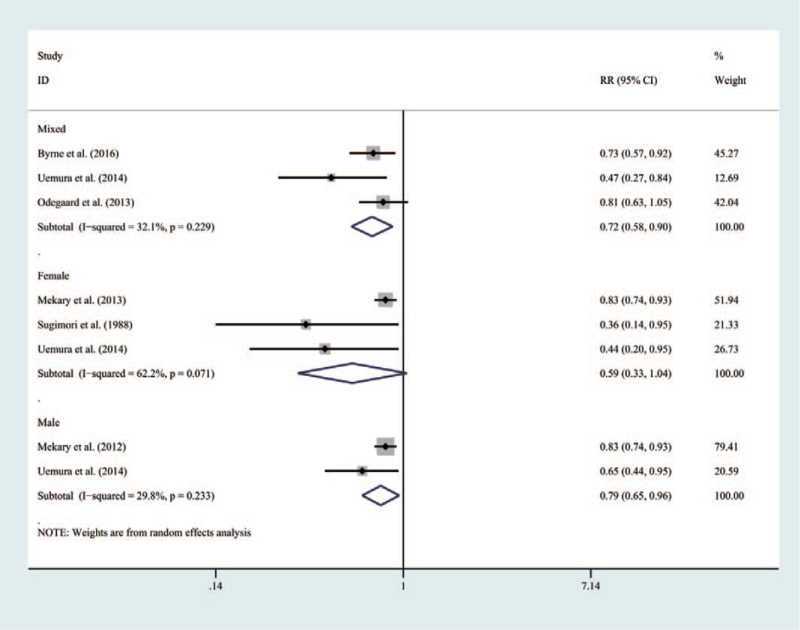
Forest map of the relationship between breakfast frequency and the risk of T2DM according to specific gender.

From the perspective of gender, as shown in Figure 3, the mixed population with a breakfast frequency_***7times/week***_ would have an obvious lower risk for T2DM than those with a frequency_***≤0∼6times/week***_ (RR = 0.72 [95% CI: 0.58–0.90], *P* = .229, *I*^2^ = 32.9%). Similarly, males with a breakfast frequency 7 times/week could have an obvious lower risk than those with a frequency_***0∼6times/week***_ (RR = 0.79 [95% CI: 0.65–0.96], *P* = .233, *I*^2^ = 29.8%). However, no significant association was found in females (RR = 0.59 [95% CI: 0.33–1.04], *P* = .071, *I*^2^ = 62.2%).

#### 3.1.2 Obesity, abdominal obesity

**Figure 4:**
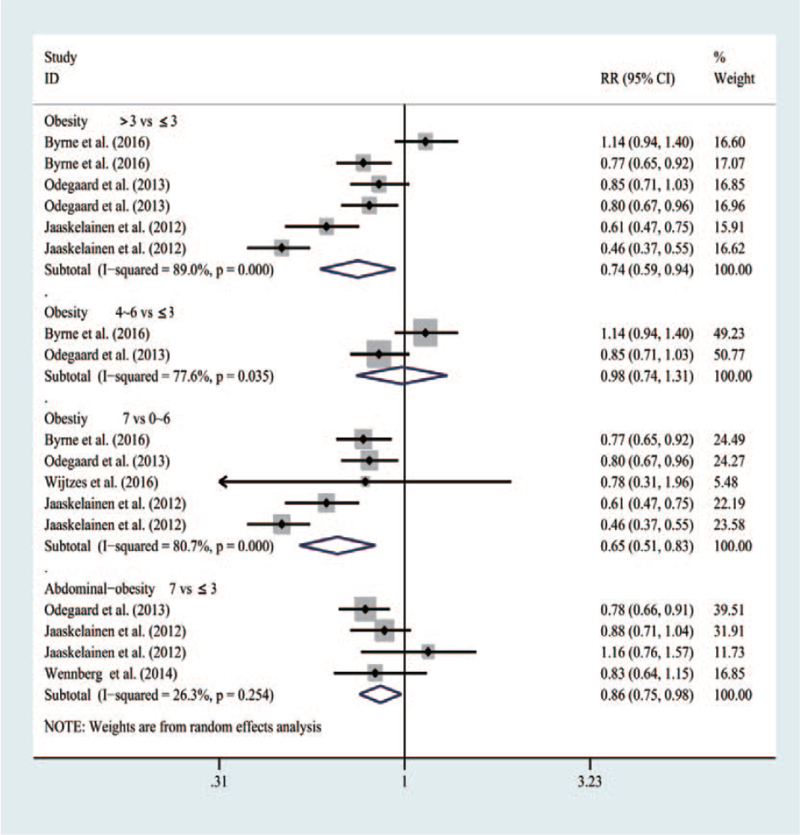
Forest map of the relationship between breakfast frequency and the risk of Obesity, Abdominal-obesity.

According to Figure 4, in five studies, 33,494 participants participated in the meta-analysis. Compared with people whose breakfast frequency was_***≤3times/week***_, those with a frequency_***>3 times/week***_ would have a decreasing risk for obesity (RR = 0.74 [95% CI: 0.59–0.94], *P* < .001, *I*^2^ = 89%). Besides, different from people who had a breakfast frequency of _***0∼6times/week***_, those with a frequency_***7times/week***_ could obviously be featured with a low risk of suffering from obesity (RR = 0.65 [95% CI: 0.51–0.83], *P* < .001, *I*^2^ = 80.7%). Nevertheless, people who had a breakfast frequency_4∼6times/week_ would not have a lower risk of getting obesity than those with a frequency_***≤3times/week***_ (RR = 0.98 [95% CI: 0.74–1.31], *P* = .035, *I*^2^ = 77.6%).

It should be noted that people who had the breakfast frequency_***7times/week***_ would have a lower risk of inducing abdominal obesity than those with a frequency_***≤3times/week***_ (RR = 0.86 [95% CI: 0.75–0.98], *P* = .254, *I*^2^ = 26.3%).

#### 3.1.3 MetS, Low HDL Cholesterolemia (LHDL-c), HC

**Figure 5:**
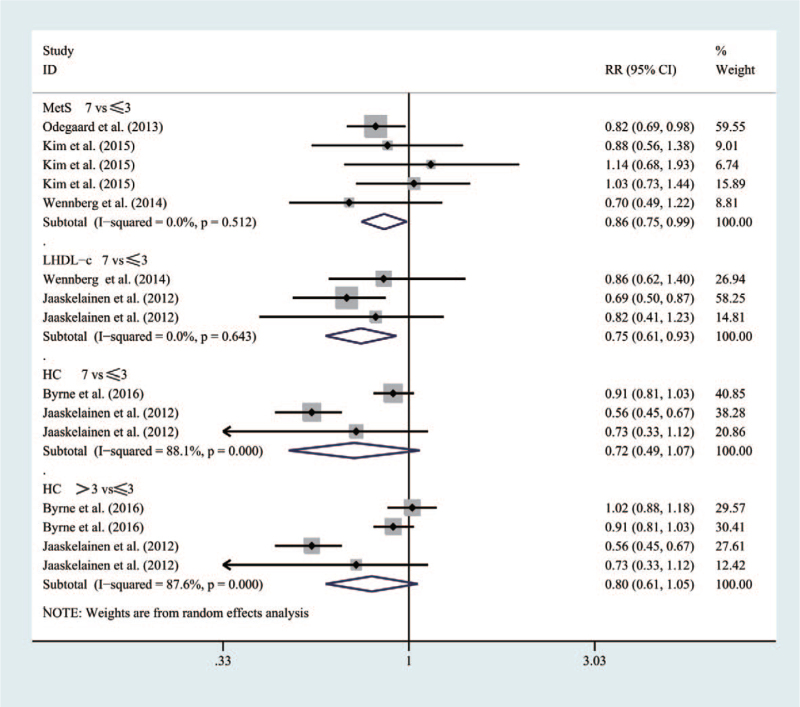
Forest map of the relationship between breakfast frequency and the risk of MetS, LHDL-c, HC.

As shown in Figure 5, five studies included 22,210 participants in the meta-analysis. Compared with people having a breakfast frequency of _***≤3times/week***_, those with a frequency_***>3 times/week***_ would be featured with the decreasing risk of MetS (RR = 0.86 [95% CI: 0.75–0.99], *P* = .512, *I*^2^ = 0%) and LHDL-c (RR = 0.75 (95% CI: 0.61–0.93], *P* = .643, *I*^2^ = 0%) accordingly.

However, for HC, people having a breakfast frequency_***7times/week***_ would not have a lower risk than those with a frequency_***≤3times/week***_ (RR = 0.72 [95% CI: 0.49–1.07], *P* < .001, *I*^2^ = 88.1%). Similarly, people with a breakfast frequency_***>3 times/week***_ could not reduce the risk of getting HC than those with a frequency_***≤*3times/week**_ (RR = 0.80 [95% CI: 0.61–1.05], *P* < .001, *I*^2^ = 87.6%).

#### 3.1.4 CVD, CHD, and CVM

**Figure 6:**
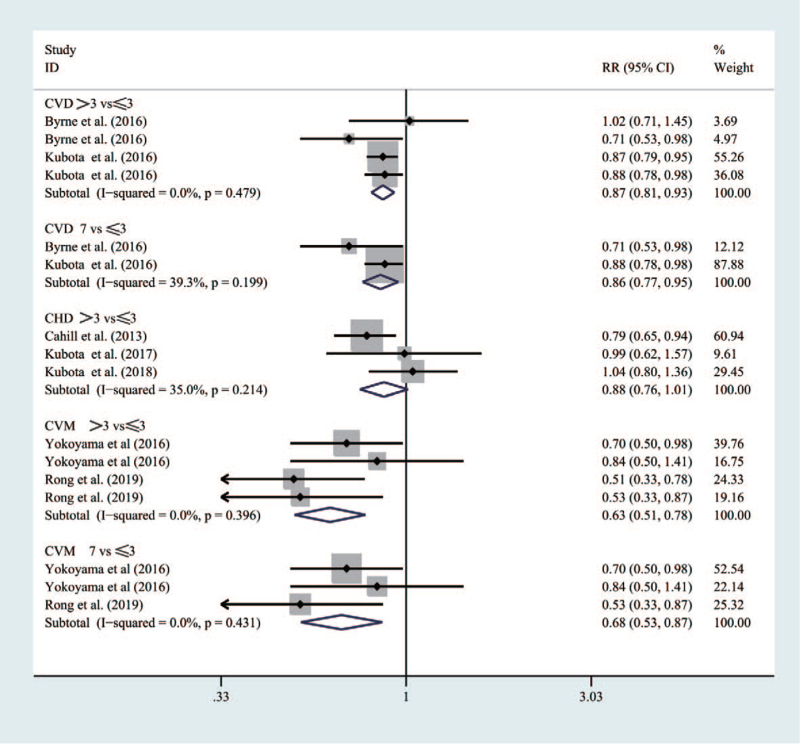
Forest map of the relationship between breakfast frequency and the risk of CVD, CHD, and CVM.

Figure 6 indicated that five studies were with 160,014 participants in the meta-analysis. Compared with people having a breakfast frequency_***≤3times/week***_, those with a frequency_***>3 times/week***_ would significantly reduce the risk of CVD (RR = 0.87 [95% CI: 0.81–0.93], *P* = .479, *I*^2^ = 0%) and CVM (RR = 0.63 [95% CI: 0.51–0.78], *P* = .396, *I*^2^ = 0%), respectively. Similarly, people with a breakfast frequency_***7times/week***_ would have an obvious lower risk than those with a frequency_***≤3times/week***_ for CVD (RR = 0.86 [95% CI: 0.77–0.95], *P* = .199, *I*^2^ = 39.3%) and CVM (RR = 0.68 [0.53–0.87], *P* = .431, *I*^2^ = 0%).

However, compared with people who had a breakfast frequency_≤***3times/week***_, those with a frequency_***>3 times/week***_ would not have a significant reducing risk for CHD (RR = 0.88 [95% CI: 0.76–1.01], *P* = .214, *I*^2^ = 35%).

#### 3.1.5 Hypertension, stroke

**Figure 7:**
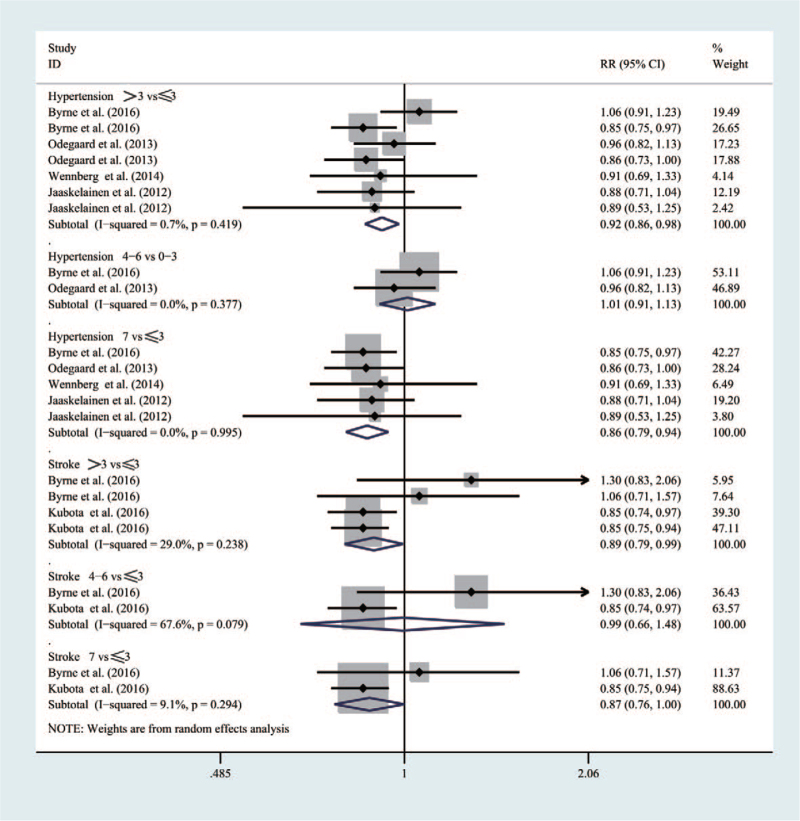
Forest map of the relationship between breakfast frequency and the risk of Hypertension, Stroke.

As shown in Figure 7, considering five studies, 103,754 participants were involved in the meta-analysis. Compared with people who had a breakfast frequency_***≤3times/week***_, those with a frequency_***>3 times/week***_ would have a lower risk of developing hypertension (RR = 0.92 [95% CI: 0.86–0.98], *P* = .419, *I*^2^ = 0.7%) and strokes (RR = 0.89 [95% CI: 0.79–0.99], *P* = .238, *I*^2^ = 29%), respectively. Similarly, people with a breakfast frequency of _***7times/week***_ would see an obvious lower risk than those with a frequency of _***≤3times/week***_ for hypertension (RR = 0.86 [95% CI: 0.79–0.94], *P* = .995, *I*^2^ = 0%) and strokes (RR = 0.87 [95% CI: 0.76–1.0], *P* = .294, *I*^2^ = 9.9%).

However, compared with people who had a breakfast frequency_***≤3times/week***_, those with a frequency_***4∼6times/week***_ could not have a significantly reducing risk of inducing hypertension (RR = 1.01 [95% CI: 0.99–1.13], *P* = .377, *I*^2^ = 0%) and strokes (RR = 0.99 [95% CI: 0.66–1.48], *P* = .079, *I*^2^ = 67.6%).

For T2DM, Figure S1 (Supplemental Digital Content) revealed the funnel plot of the comparison between higher breakfast frequency and lower breakfast frequency. Besides, Begg's test suggested that no significant publication bias was observed (*P* ***=*** .373). According to Figure S2 (Supplemental Digital Content), the sensitivity analysis showed that the pooled results changed slightly after each study was removed one by one.

For strokes, the funnel plot of the comparison between higher breakfast frequency and lower breakfast frequency was displayed in Figure S3 (Supplemental Digital Content). Besides, Begg's test revealed no obvious evidence of a publication bias (*P* ***=*** .929). As shown in Figure S4 (Supplemental Digital Content), from sensitivity analysis, it could be found that after each study was eliminated in sequence, the summary results changed slightly.

As for other specific cardiovascular or metabolic diseases, sensitivity and subgroup analysis could not be conducted due to the limited number of current studies.

## 4 Discussion

In this meta-analysis, 15 cohort studies were included, with 417,093 participants being involved, and it was indicated that regular breakfast habits (7 times/week) could significantly reduce the occurrence of cardiovascular and metabolic diseases, as well as such specific-diseases as T2DM, obesity, hypertension, strokes, hypercholesterolemia, MetS and abdominal obesity. Moreover, regular breakfast habits (7 times/week) yielded the maximum potential cardio-metabolic benefits, and even skipping breakfast once per week might reduce the benefits for T2DM, obesity, hypertension and strokes to a great extent. However, there was no significant correlation between regular breakfast habits and the occurrence of hypercholesterolemia. Besides, when stratified by gender, no significant correlation can be seen between regular breakfast habits and the risk for T2DM in females.

Breakfast is taken as the most important meal of a day, and irregular breakfast habits were associated with the occurrence of various cardiovascular and metabolic diseases. Several meta-analyses^[39–42]^ have revealed breakfast skipping was closely related to the occurrence of T2DM or obesity. However, most of these studies were cross-sectional, which might weaken the evidence value when being compared with cohort studies. Moreover, few studies have focused on the relationship between breakfast and other specific cardio-metabolic diseases. Diet patterns and physical activity go hand in hand with human health. Recently, Kwok et al^[43]^ have comprehensively investigated the relationship between dietary components and cardiovascular mortality, and then they found many dietary components appear to be beneficial to cardiovascular disease and mortality, including fish, grains, vegetables and nuts, but tinned fruit and processed meat seem to be harmful. Musse et al^[44]^ pointed out that within 30 days after hospital discharge, the association between skipping breakfast concomitant and late-night dinner increased the likelihood of death, reinfarction, and postinfarction angina four to five times. Simultaneously, Cheng et al^[45]^ systematically analyzed the relationship between physical activity and cardiovascular mortality, and it was shown that leisure-time physical activity shows a linearly negative correlation with the risk of cardiovascular mortality. In a sense, the current meta-analyses have enriched previous studies.

In the long process of human evolution, regular eating not only keeps the body at a stable rhythm, but also gradually forms a specific circadian rhythm system and the internal clock, whereas it has been reported that irregular breakfast is a key factor affecting the biological clock.^[46,47]^ Besides, the effects of breakfast frequency on cardio-metabolic diseases might be explained by the following potential mechanisms. First, irregular breakfast is deemed to be a sign of unhealthy eating patterns and lifestyles, which might be a long-term behavior from childhood to adulthood.^[48,49]^ Recently, several studies^[50–52]^ have indicated that irregular breakfast can significantly decrease satiety, thus leading people to eat more at lunch, which will further increase the production of hunger-related hormones that are associated with higher glucose responses and obesity. Secondly, breakfast eating behavior may have a mediating effect on subsequent metabolic outcomes. Studies conducted by Wennberg et al and Nas et al^[53,54]^ have shown that irregular breakfast could result in inflexibility in the metabolic system, thus causing an increase in postprandial hyperglycemia and fat oxidation, while the release rhythm of insulin in the body would not be altered, thus resulting in a low inflammatory state and the impaired blood glucose regulation system. Besides, the study by Myers C et al.^[55]^ showed that the consumption of nutritionally matched fruit smoothie at breakfast did not affect acute dietary intake, yet the effects of consuming fruit smoothie instead of cereal for breakfast on body weight and health biomarkers to be further clarified. Similarly, the study by Rosi A et al^[56]^ showed that food patterns at lunch did not alter after consuming different categories of breakfast, and further work is needed on the effect on the amount of food consumed at lunch.

Thirdly, the study conducted by Uzhova et al^[57]^ suggested that irregular breakfast habits might have an impact on lipid levels, increase atherosclerotic LDL levels, and further cause atherosclerosis that leaded to cardiovascular disease, which was similar to our conclusions. However, no relationship was found between breakfast and hypercholesterolemia in our meta-analysis, which might be related to the current high-energy dietary structure similar to the results of a recent national survey by Jung et al^[58]^ suggesting that lower breakfast frequency was related with metabolic syndrome in men, yet there is no significant association in women. Recently, Yao et al^[59]^ conducted a meta-analysis of the effect of fatty acid composition in breakfast on postprandial lipids, and the results showed that triglyceride after breakfast was not significantly altered regardless of saturated or unsaturated fatty acid composition. However, when a subgroup analysis was performed with 8 h as the cut-off, it was shown that triglyceride levels were decreased until 8 h after the ones having breakfast with saturated acid composition, while triglyceride levels were increased after 8 h after after those having breakfast with unsaturated acid composition. In addition, there may be a cumulative temporal relationship between irregular breakfast behavior and subsequent disease onset, and more research is needed to further explain these complex mechanisms. Fourthly, skipping breakfast can also affect the regulation of hypothalamic-pituitary-adrenal axis on blood pressure, further causing hypertension in the morning.^[60,61]^

Based on the current meta-analysis, this paper has the following advantages. First, this is the first systematic analysis on specific metabolic diseases, and all related specific diseases were reported in the original study. Secondly, four electronic databases were retrieved, and previous meta-analyses were reviewed to ensure the most comprehensive inclusion of the studies. In addition, all studies mentioned here were cohort ones, which guaranteed the evidence value. Thirdly, the included studies were featured with large sample size and high quality.

Inevitably, the present meta-analysis possesses the following limitations. First, although most studies have adjusted the maximum mixed variable, the influence of residual confounding factors could not be excluded. Secondly, due to the limited number of current studies, sensitivity and subgroup analysis could not be conducted for explaining the high heterogeneity among several studies. Thirdly, in most of the studies, questionnaires were adopted to record the frequency of breakfast. In this case, the influence of subjective factors on the results of the studies could not be ruled out. Finally, as the included studies were mainly limited to Asia, Europe and the Americas, the impact on other regions remains unknown.

## 5 Conclusions

It can be concluded that daily eating habits would bring the greatest cardio-metabolic benefits, reducing the risk of T2DM, obesity, hypertension, etc, yet not be significantly related to hypercholesterolemia.

## Author contributions

ZH Li, LX, and RD have contributed equally to this work.

Zhi-Hui Li had full access to all of the data in the study and takes responsibility for the integrity of the data and the accuracy of the data analysis.

**Acquisition, analysis, or interpretation of data**: zhi-hui, li-jie and Wang.

**Administrative, technical, or material support**: zhi-hui.

**Concept and design:** zhi-hui, xu, and Wang.

**Conceptualization:** Zhi-hui Li, lei Xu.

**Critical revision of the manuscript for important intellectual content**: All authors.

**Data curation:** Zhi-hui Li, li-jie Li.

**Drafting of the manuscript**: All authors.

**Formal analysis:** Zhi-hui Li, lei Xu, rao dai, li-jie Li, haojie wang.

**Funding acquisition:** rao dai.

**Investigation:** lei Xu, li-jie Li.

**Methodology:** Zhi-hui Li, lei Xu, haojie wang.

**Obtained funding**: None.

**Project administration:** lei Xu.

**Resources:** rao dai.

**Statistical analysis**: Li, liu.

**Supervision:** rao dai, zhi-hui.

**Writing – original draft:** Zhi-hui Li.

**Writing – review & editing:** Zhi-hui Li.

## Supplementary Material

Supplemental Digital Content

## Supplementary Material

Supplemental Digital Content

## Supplementary Material

Supplemental Digital Content

## Supplementary Material

Supplemental Digital Content

## Supplementary Material

Supplemental Digital Content
