# Supplementary material for: Effects of regular breakfast habits on metabolic and cardiovascular diseases: A protocol for systematic review and meta-analysis
Source: Medicine (Baltimore). 2021 Nov 5;100(44):e27629. doi: 10.1097/MD.0000000000027629 (PMC8568444; doi:10.1097/MD.0000000000027629)
Supplement: Supplemental Digital Content [file medi-100-e27629-s001.docx]

***[Appendix](javascript:;) 1***

***Pubmed***

1. **Search ((((((((("Breakfast"[Mesh]) OR Breakfasts[Title/Abstract]) OR Morning Meal[Title/Abstract]) OR Breakfast Time[Title/Abstract]) OR Breakfast Times[Title/Abstract]) OR Time, Breakfast[Title/Abstract]) OR Times, Breakfast[Title/Abstract]) OR Meal, Morning[Title/Abstract]) OR Meals, Morning[Title/Abstract]) OR Morning Meals[Title/Abstract]**
2. **Search (Cardiac[Title/Abstract]) OR cardiovascular[Title/Abstract]**
3. **Search ((((("Metabolic Diseases"[Mesh]) OR Disease, Metabolic) OR Thesaurismosis) OR Metabolic Disease) OR Thesaurismoses) OR Diseases, Metabolic**
4. **2 or 3**
5. **1 and 4 1259**

***Embase***

**1. 'meal'/exp**

**2. breakfast:ab,ti OR lunch:ab,ti OR 'standard meal':ab,ti OR meals:ab,ti**

**3. #1 OR #2**

**4. 'cardiovascular disease'/exp**

**5. angiocardiopathy:ab,ti OR 'angiocardiovascular disease':ab,ti OR 'cardiovascular complication':ab,ti OR 'cardiovascular diseases':ab,ti OR 'cardiovascular disorder':ab,ti OR 'cardiovascular disturbance':ab,ti OR 'cardiovascular lesion':ab,ti OR 'cardiovascular syndrome':ab,ti OR 'cardiovascular vegetative disorder':ab,ti OR 'complication, cardiovascular':ab,ti OR 'disease, cardiovascular':ab,ti OR 'major adverse cardiovascular event':ab,ti**

**6. #4 or #5**

**7. 'metabolic disorder'/exp**

**8. 'metabolic disease':ab,ti OR 'metabolic diseases':ab,ti OR 'metabolic disturbance':ab,ti OR 'metabolic error':ab,ti OR 'metabolism disorder':ab,ti OR (nutritional:ab,ti AND 'metabolic diseases':ab,ti) OR 'water-electrolyte imbalance':ab,ti**

**9. #7 OR #8**

**10. #6 OR #9**

**11. #3 AND #10 1509**

***Cochrane***

1. **MeSH descriptor: [Breakfast] explode all trees**
2. **(Morning Meals):ti,ab,kw OR (Breakfasts):ti,ab,kw OR (Breakfast Time):ti,ab,kw OR (Meal, Morning):ti,ab,kw OR (Morning Meal):ti,ab,kw**
3. **(Meals, Morning):ti,ab,kw OR (Time, Breakfast):ti,ab,kw OR (Breakfast Times):ti,ab,kw OR (Times, Breakfast):ti,ab,kw**
4. **#1 or #2 or #3**
5. **MeSH descriptor: [Cardiovascular Diseases] explode all trees**
6. **(Diseases, Cardiovascular):ti,ab,kw OR (Cardiovascular Disease):ti,ab,kw OR (Disease, Cardiovascular):ti,ab,kw**
7. **#5 or #6**
8. **MeSH descriptor: [Metabolic Diseases] explode all trees**
9. **(Thesaurismoses):ti,ab,kw OR (Diseases, Metabolic):ti,ab,kw OR (Metabolic Disease):ti,ab,kw AND (Thesaurismosis):ti,ab,kw AND (Disease, Metabolic):ti,ab,kw**
10. **#8 or #9**
11. **#7 or #10**
12. **#4 and #11 827**

***Web of science***

1. **ALL=( Breakfast OR Breakfasts OR Breakfast Time OR Breakfast Times OR Time, Breakfast OR Times, Breakfast OR Morning Meal OR Meal, Morning OR Meals, Morning OR Morning Meals)**
2. **ALL=( cardiovascular OR cardiac)**
3. **ALL=( Metabolic Diseases OR Disease, Metabolic OR Metabolic Disease OR Thesaurismosis OR Thesaurismoses OR Diseases, Metabolic)**
4. **2 or 3**
5. **1 and 4 1154**
