# Supplementary material for: Effects of regular breakfast habits on metabolic and cardiovascular diseases: A protocol for systematic review and meta-analysis
Source: Medicine (Baltimore). 2021 Nov 5;100(44):e27629. doi: 10.1097/MD.0000000000027629 (PMC8568444; doi:10.1097/MD.0000000000027629)
Supplement: Supplemental Digital Content [file medi-100-e27629-s003.doc]

***Supplementary Figure 2.*** Sensitivity analysis of the comparison between higher breakfast frequency and lower breakfast frequency for T2DM

***
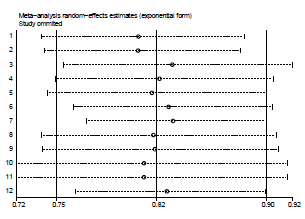
***
