# Supplementary material for: Effects of regular breakfast habits on metabolic and cardiovascular diseases: A protocol for systematic review and meta-analysis
Source: Medicine (Baltimore). 2021 Nov 5;100(44):e27629. doi: 10.1097/MD.0000000000027629 (PMC8568444; doi:10.1097/MD.0000000000027629)

***Supplementary Figure 4.*** Sensitivity analysis of the comparison between higher breakfast frequency and lower breakfast frequency for for strokes


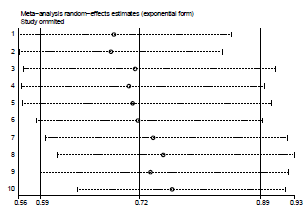

Supplement: Supplemental Digital Content [file medi-100-e27629-s005.doc]
